# Usefulness of serum HE4 in endometriotic cysts

**DOI:** 10.1038/sj.bjc.6605119

**Published:** 2009-06-09

**Authors:** M Montagnana, G Lippi, E Danese, M Franchi, G C Guidi

**Affiliations:** 1 Dipartimento di Scienze Morfologico-Biomediche, Sezione di Chimica Clinica, Università di Verona, Verona, Italy; 2 Unità di Ostetricia, Dipartimento Materno ed Infantile, Università di Verona, Verona, Italy

**Sir**,

Huhtinen *et al* (2009) recently reported about the usefulness of Human Epididymal Protein 4 (HE4) in discriminating ovarian tumours from ovarian endometriotic cysts, which is of pivotal importance as CA125 is often increased in benign gynaecological diseases such as endometriosis (Muyldermans *et al*, 1995). Therefore, we performed a similar study on 46 patients affected by ovarian cancer and 21 patients affected by endometriosis (12 ovarian endometrioma and 9 non-ovaric endometriosis). Serum levels of CA125 were determined using a chemiluminescent enzyme immunoassay on the Liaison (DiaSorin, Saluggia, Italy). Serum levels of HE4 were determined using an ELISA kit developed by Fujirebio Diagnostic Inc. (Malvern, PA, USA).

The mean serum concentration of HE4 in patients with ovarian cancer was significantly higher than that observed in patients with endometriosis (810.0 *vs* 49.4 pM, *P*<0.0001). The mean serum concentrations of HE4 in our patients with ovarian endometrioma were similar to those observed in the study of Huhtinen *et al* (2009) (47.5 *vs* 46.0 pM). However, in our investigation, 10 out of 12 patients with ovarian endometrioma showed CA125 values higher than 35 U ml^−1^, but only one patient showed an HE4 serum concentration higher than 70 pM, which is the upper limit of the reference range (Moore *et al*, 2008). More importantly, receiver operator characteristic curve analysis showed that HE4 has a significantly higher area under the curve as compared with CA125 (0.85 *vs* 0.77, *P*<0.0001) for differentiating ovarian cancer from ovarian endometrioma. The results of our investigation confirm that HE4 is a promising marker for the early differentiation of ovarian cancer from ovarian endometriotic cysts, showing better diagnostic performances than CA125.
